# ﻿*Notomastusbermejoi*, a new species of Capitellidae (Annelida, Polychaeta) from the Gulf of California, with morphological remarks on species with hooks in thoracic chaetigers

**DOI:** 10.3897/zookeys.1102.83198

**Published:** 2022-05-19

**Authors:** Pablo Hernández-Alcántara, María E. García-Garza, Vivianne Solís-Weiss

**Affiliations:** 1 Unidad Académica de Ecología y Biodiversidad Acuática, Instituto de Ciencias del Mar y Limnología, Universidad Nacional Autónoma de México, Circuito Exterior S/N, Cd. Universitaria, Cd. de México, 04510, Mexico Universidad Nacional Autónoma de México Mexico City Mexico; 2 Universidad Autónoma de Nuevo León, Facultad de Ciencias Biológicas, Laboratorio de Biosistemática, Apartado Postal 5 “F”, San Nicolás de los Garza, Nuevo León, Mexico Universidad Autónoma de Nuevo León San Nicolás de los Garza Mexico; 3 Unidad Académica de Sistemas Arrecifales, Instituto de Ciencias del Mar y Limnología, Universidad Nacional Autónoma de México, Prol. Av. Niños Héroes s/n Puerto Morelos Quintana Roo, 77580, Mexico Universidad Nacional Autónoma de México Puerto Morelos Mexico

**Keywords:** Mexican Pacific, new species, Polychaeta, staining patterns, taxonomy

## Abstract

*Notomastusbermejoi***sp. nov.** from the Gulf of California shelf is described, illustrated, and compared with its congeners bearing hooded hooks in thoracic chaetigers. This new species is characterized by the presence of a prostomial palpode, only notopodia in the first chaetiger, hooded hooks in neuropodia of chaetiger 11, and its distinct methyl green staining pattern consisting of: chaetigers 1–4 slightly stained, chaetigers 5–10 with green bands encircling the segments, and a darker, solid, green band encircling the body in chaetigers 11–12. It is mainly distributed in the central Gulf of California in fine sand bottoms (62–96%) at 32–106.4 m depth, tolerating a wide range of temperature (13.2–17.59 °C), dissolved oxygen (0.8–4.93 ml/L), and organic carbon (3.0–7.2%). The type material and original descriptions of *Notomastus* species with hooks in thoracic chaetigers were examined; an identification key and tables with morphological distinctive characteristics, methyl green staining patterns, and geographic distribution of these close species are provided.

## ﻿Introduction

Capitellids are burrowing worms, usually elongate and thread-like. They are among the most frequently recorded polychaetes in marine soft bottoms, living at a wide bathymetric range from intertidal to deep sea and may even be the dominant organisms in infaunal communities, especially in organically enriched sediments (Blake 2000). The family Capitellidae Grube, 1862 is one of the oldest recognized polychaete families and currently is composed of approximately 200 valid species belonging to 43 genera (Magalhães and Bailey-Brock 2012; Silva and Amaral 2019). Although they can be easily recognized at the family level, their accurate identification, even at the generic level, is difficult since very few distinctive morphological characters are visible (Magalhães and Blake 2019). So, definitions at the generic and species level have always been controversial, as they are mainly based on the number and structure of the thoracic chaetigers and the distribution of different types of chaetae along the body. However, as demonstrated recently (Magalhães and Blake 2019), those characters, far from being stable, can change with age and sexual maturity and, thus, 21 genera are monotypic and most of them are represented by a single type specimen, frequently incomplete.

The genus *Notomastus* M. Sars, 1851 is characterized by a thorax with 12 segments: the peristomium and 11 chaetigers; the first thoracic chaetiger is uni- or biramous and the last may have capillary chaetae, hooded hooks, or a mixture of both; in the abdominal chaetigers, hooded hooks are present, while branchiae can be present or absent (García-Garza and de León-González 2015). Ten of the 44 valid species, including *Notomastusbermejoi* sp. nov., were originally described from the Gulf of California: *N.abyssalis* Fauchald, 1972, *N.angelicae* Hernández-Alcántara and Solís-Weiss, 1998, *N.cinctus* Fauchald, 1972, *N.fauchaldi* García-Garza and de León-González, 2015, *N.landini* García-Garza and de León-González, 2015, *N.lobulatus* García-Garza and de León-González, 2015, *N.mazatlanensis* García-Garza, de León-González and Tovar-Hernández, 2019, *N.precocis* Hartman, 1960, and *N.sonorae* Kudenov, 1975.

Predictably, in *Notomastus*, the taxonomic problems detected in other capitellid genera also occur, with several species that do not entirely fit the genus definition, e.g., *N.exsertilis* Saint-Joseph, 1906 has only 10 thoracic chaetigers and bears capillary chaetae in the first two abdominal segments, or *N.hedlandica* where capillary chaetae in the first abdominal segment are present (Hartmann-Schröder 1979), making us think that they probably belong to other genera (García-Garza et al. 2019).

This confusing situation usually leads to misidentifications. This is the case of *Notomastusamericanus* Day, 1973, which was originally described from off Beaufort, North Carolina, and then reported from the Gulf of California by Hernández-Alcántara and Solís-Weiss (1993, 1998, 1999), due to the presence of hooded hooks in neuropodia of chaetiger 11. However, *N.americanus* was later synonymized with *Notomastushemipodus* Hartman, 1945 by García-Garza et al. (2012), based on their revision of the type material. Then, recently, the careful taxonomic examination of the specimens catalogued as *N.americanus* deposited in the Colección Nacional de Anélidos Poliquetos, Instituto de Ciencias del Mar y Limnología (ICML)Universidad Nacional Autónoma de México (UNAM), Mexico City, revealed significant differences, not only with the type material of *N.americanus* (= *N.hemipodus*) but also with close species. That is why the aim of this study is to describe a new species of *Notomastus* from those misidentified organisms. To corroborate the status of the new species, we also reviewed the type material and original descriptions of *Notomastus* species with hooded hooks in thoracic chaetigers deposited in the National Museum of Natural History, Smithsonian Institution and Natural History Museum of Los Angeles County. An identification key and tables with morphological distinctive characteristics and methyl green staining patterns to support the future identification of these capitellids are provided.

## ﻿Materials and methods

**Figure 1.**
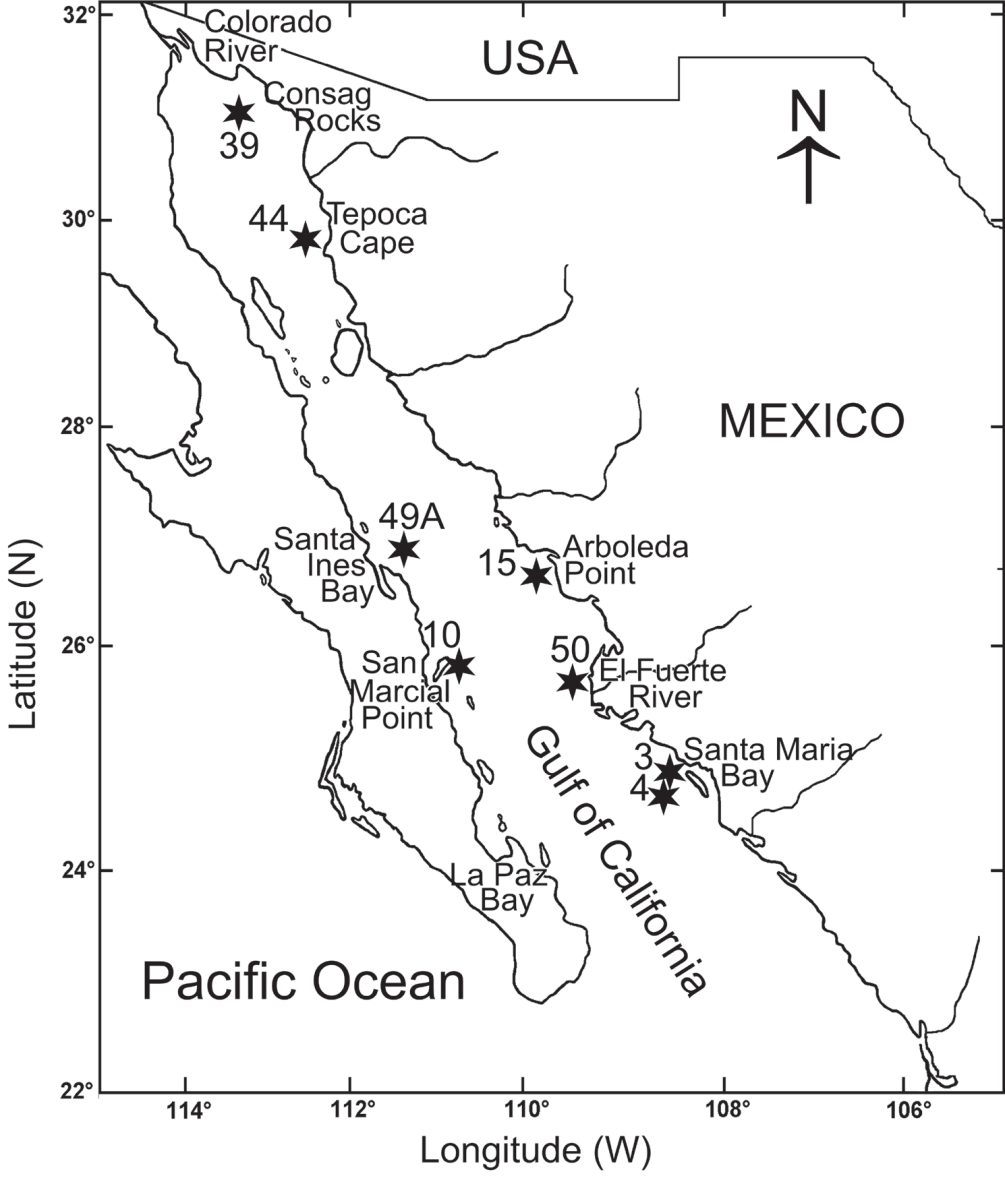
Gulf of California showing the sampling stations where *Notomastusbermejoi* sp. nov. was collected.

The material examined was collected in the continental shelf of the Gulf of California, Mexican Pacific (20°30'–31°38'N, 105°42'–114°50'W), as part of the oceanographic expedition “Cortes 2” (Fig. 1; Table 1) on board the R/V *El Puma* of the Universidad Nacional Autónoma de México (**UNAM**). The samples were collected with a Smith-McIntyre grab (0.1 m^2^) and sieved through a 0.5 mm mesh. The specimens were fixed in 10% formalin in seawater and later preserved in 70% ethanol. Additionally, at each station, depth, temperature, and salinity were measured with a Niels Brown CTD, and the dissolved oxygen determined by the Winkler method (Strickland and Parsons 1972). The organic matter content was evaluated by the Walkley and Black (1934) acid digestion method and the sediment texture was determined following the method of wet sieving (Folk 1980).

**Table 1.** Location and environmental conditions of the sampling stations where *Notomastusbermejoi* sp. nov. was collected.

Station	GPS Coordinates	Depth (m)	Salinity (psu)	Temperature (°C)	Dissolved oxygen (ml/lt)	Organic matter (%)	Sand (%)
39	30°59.4'N, 114°04.1'W	106.4	35.16	13.2	1.73	3.0	82
44	30°02.4'N, 112°55.4'W	104.1	35.26	14.2	2.40	7.2	74
15	26°51.1'N, 110°06.5'W	49.8	35.22	14.1	1.04	4.6	88
50	25°46.8'N, 109°35.4'W	97.0	34.99	13.2	1.47	5.7	62
3	25°02.4'N, 108°31.7'W	32.0	35.04	14.0	1.02	5.7	96
4	24°56.9'N, 108°41.8'W	79.0	35.00	13.2	0.80	3.0	77
49A	26°59.6'N, 111°50.4'W	100.0	35.10	13.2	1.34	3.6	96
10	25°58.6'N, 111°06.9'W	39.0	35.51	17.5	4.93	4.1	87

The specimens were examined under dissecting and compound light microscopes, both with an integrated camera for photography. Detailed examination of the chaetal types, distribution, and morphology were supported with scanning electron microscope images: specimens were dehydrated via a graded ethanol series, critical-point dried with liquid CO_2_, coated with gold, and examined in a JEOL JSM6360LV microscope at the Instituto de Ciencias del Mar y Limnología (**ICML**), UNAM. The methyl green staining pattern was examined by immersing the specimens for 2 min in a saturated solution of methyl green in 70% ethanol, then washing them in ethanol 70% to remove the excess methyl green (Warren et al. 1994).

All identified specimens and the type material of the new species were deposited in the Colección Nacional de Anélidos Poliquetos of the ICML, UNAM (CNAP-ICML: DFE.IN.061.0598). Paratypes were deposited in the Natural History Museum of Los Angeles County (**LACM-AHF Poly**).

The type material of *Notomastus* species with hooded hooks in thoracic chaetigers deposited in the National Museum of Natural History, Smithsonian Institution (**USNM**) and in the Natural History Museum of Los Angeles County were also examined to compare their morphological characteristics with those found in the new capitellid.

## ﻿Results

### ﻿Taxonomy

**Family Capitellidae Grube, 1862**

#### ﻿Genus Notomastus

Taxon classificationAnimaliaCapitellidaCapitellidae

M. Sars, 1851

B7799DF9-6A60-5E8F-BAAA-D544A465F042

##### Type species.

*Notomastuslatericeus* M. Sars, 1851: 199–200.

##### Diagnosis.

The genus *Notomastus* has a conical prostomium, palpode present or absent; eyespots present in multiple spots or absent. Peristomium clearly distinct from prostomium. First chaetiger uniramous or biramous. Eleven thoracic chaetigers. Chaetigers 1–11 with only capillaries or last 1–3 thoracic chaetigers with notopodial capillaries and neuropodial hooks. Abdominal segments with only hooded hooks. Branchiae present or lacking. Genital pores present or absent. Lateral organs present on thorax and abdomen. Pygidium unadorned but unknown for many species (Magalhães and Blake 2019).

#### ﻿ Notomastus bermejoi sp. nov.

Taxon classificationAnimaliaCapitellidaCapitellidae

365EC32B-DB60-5BAB-ABE6-E3C49CE0A4BD

http://zoobank.org/8BDAB03F-3774-4BF4-80E6-514FF76B5E96

Figs 2A–J , 3A–F

Notomastus americanus —Hernández-Alcántara and Solís-Weiss 1993: 1034, 1998: 710–711, 1999: 27.
not Notomastusamericanus—Day 1973: 100, fig. 131n (= N.hemipodus Hartman, 1945 fide García-Garza et al. 2012).

##### Material examined.

**Type locality.** Mexico • Gulf of California, Tepoca Cape; 30°02.4'N, 112°55.4'W; 104.1 m. ***Holotype***: from type locality; 17 Mar. 1985; P. Hernández-Alcántara leg.; fine sand sediment; CNAP-POH-17-002. ***Paratypes***: Mexico • 2 specs.; Gulf of California; same collection data as for holotype; CNAP-POP-005 • 2 specs.; El Fuerte River, Sta. 50; 25°46.8'N, 109°35.4'W; 87 m; 20 Mar. 1985; same collector as for preceding; fine sand sediment; CNAP-POP-006 • 1 spec.; San Marcial Point, Sta. 10; 25°58.6'N, 111°06.9'W; 39 m; 11 Mar. 1985; same collector as for preceding; fine sand sediment; CNAP-POP-17-007 • 1 spec.: Arboleda Point, Sta. 15; 26°51.1'N, 110°06.5'W; 49.8 m; 12 Mar. 1985; same collector as for preceding; fine sand sediment; coated with gold for SEM studies; CNAP-POP-17-008 • 4 specs.; North Consag Rocks, Sta. 39; 30°59.4'N, 114°04.1'W; 106.4 m; 16 Mar. 1985; same collector as for preceding; fine sand sediment; LACM-AHF Poly 12858.

##### Additional material.

Mexico • 1 spec.; Gulf of California, El Fuerte River, Sta. 50; 25°46.8'N, 109°35.4'W; 87 m; 20 Mar. 1985; same collector as for preceding; CNAP-PO-036/GCA-CS-2006 • 1 spec.; San Marcial Point, Sta. 10; 25°58.6'N, 111°06.9'W); 39 m; 11 Mar. 1985; same collector as for preceding; CNAP-PO-036/GCA-CS-2007 • 3 specs.; Arboleda Point, Sta. 15; 26°51.1'N, 110°06.5'W; 49.8 m; 12 Mar. 1985; same collector as for preceding; CNAP-PO-036/GCA-CS-2008 • 1 spec.; Santa Maria Bay, Sta. 3; 25°02.4'N, 108°31.7'W; 32 m; 19 Mar. 1985; same collector as for preceding; CNAP-PO-036/GCA-CS-2009 • 6 specs.; North Consag Rocks, Sta. 39; 30°59.4'N, 114°04.1'W; 196.4 m; 16 Mar. 1985; same collector as for preceding; CNAP-PO-036/GCA-CS-2010 • 2 specs.; Santa Maria Bay, Sta. 4; 24°56.9'N, 108°41.8'W; 79 m; 10 Mar. 1985; same collector as for preceding; CNAP-PO-036/GCA-CS-2011 • 1 spec.; Santa Ines Bay, Sta. 49A; 26°59.6'N, 111°50.4'W; 100 m; 19 Mar. 1985; same collector as for preceding; CNAP-PO-036/GCA-CS-2012.

##### Comparative type material examined.

*Notomastusamericanus* Day, 1973. ***Holotype***: USA • 1 spec.; North Carolina, Beaufort; 4 Jun. 1965; USNM 43118. ***Paratypes***: USA • 14 specs.; same collection data as for holotype; USNM 43119.

*Notomastusangelicae*—Hernández-Alcántara and Solís-Weiss, 1998. ***Holotype***: Mexico • 1 spec.; Gulf of California, El Fuerte River; 25°39.8'N, 109°28.5'W; 28.6 m; 20 Mar. 1985; USNM 180697. ***Paratypes***: Mexico • 5 spec.; same collection data as for holotype; LACM-AHF-POLY-1902 • 5 specs.; same collection data as for holotype; USNM 180698.

*Notomastusdaueri* Ewing, 1982. ***Holotype***: USA • 1 spec.; Louisiana, Northern Gulf of Mexico; 28°56'N, 90°04'W; 27.7 m; 16 Apr. 1980; USNM 71442. ***Paratype***: USA • 1 spec.; same locality as for holotype; 21 Aug. 1980; USNM 71443.

*Notomastusprecocis* Hartman, 1960. ***Holotype***: USA • 1 spec.; Santa Catalina Basin, California, Sta. 2848; 33°18.0'N, 118°42.0'W; 1305 m; 23 Jun. 1954; LACM-AHF POLY 0416.

*Notomastusteres* Hartman, 1965. ***Holotype***: Bermuda • 1 spec.; Bermuda, Sta. 2; 32°16.5'N, 64°36.3'W; 1700 m; 18 Apr. 1960; LACM AHF 0418. ***Paratypes***: Bermuda • 1 spec.; same collection data as for holotype; LACM AHF 0418 • 1 spec.; same collection data as for holotype; USNM 57105.

##### Etymology.

The species is named after the Bermejo Sea, as the Gulf of California was originally known, and where this new capitellid was collected.

##### Diagnosis.

Prostomium conical with anterior palpode. Peristomium and first six chaetigers with tessellated epithelium. Thorax with peristomium and 11 chaetigers; first chaetiger uniramous. Chaetiger 1–10 with only bilimbate capillaries, chaetiger 11 with notopodial bilimbate capillaries and neuropodial hooded hooks. Thoracic and abdominal chaetigers biannulate. Transition between thorax and abdomen marked by chaetal change. Methyl green staining pattern consisting of: chaetigers 1–4 slightly stained, chaetigers 5–10 with green bands encircling the segments, and a darker, solid, green band encircling the body in chaetigers 11 and 12. Abdominal chaetigers with hooded hooks in both rami. Notopodial and neuropodial abdominal hooded hooks of similar shape. Branchiae not observed. Pygidium unknown.

##### Description.

**Figure 2.**
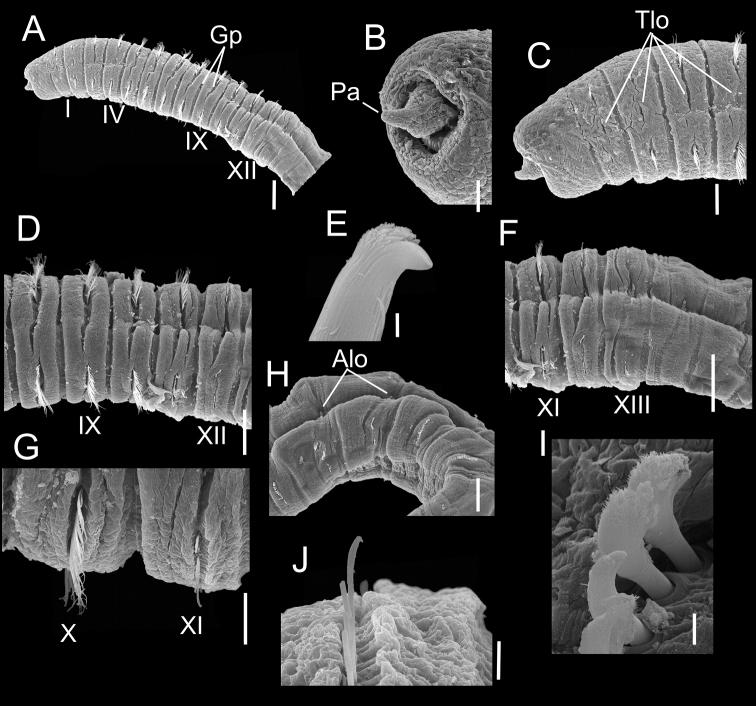
*Notomastusbermejoi* sp. nov., paratype (CNAP-POP-17-008) **A** thoracic region, lateral view **B** prostomium, frontal view **C** prostomium and chaetigers 1–4, lateral view **D** chaetigers 8–12, lateral view **E** hooded hook chaetiger 11 **F** chaetigers 11–14, lateral view **G** neuropodia 10–11 **H** abdominal chaetigers (18–21) **I** abdominal hooded hooks **J** neuropodia 11, hooded hooks. Abbreviations: Alo = abdominal lateral organs; Gp = genital pores; Pa = palpode; Tlo = thoracic lateral organs. Scale bars: 500 μm (**A**); 100 μm (**B, G**); 200 μm (**C, D, F, H**); 2 μm (**E**); 5 μm (**I**); 20 μm (**J**).

Holotype incomplete, with 32 segments, 13.5 mm long, 0.8 mm wide. Paratypes incomplete, with 18–40 segments, 6.5–16.5 mm long, 0.7–0.8 mm wide. Colour in ethanol light brown. Prostomium conical, with anterior palpode (Fig. 2A, B). Proboscis with soft papillae basally, smooth surface distally. Peristomium and chaetigers 1–5 or 1–6 with tessellated epithelium (Fig. 2C), following thoracic segments smooth (Fig. 2A). Thorax with 12 segments, including peristomium and 11 biannulate chaetigers with deep intra- and intersegmental grooves (Fig. 2A, C, D). First chaetiger uniramous with only notopodial capillaries (Fig. 2C), chaetigers 2–10 with only bilimbate capillaries in both rami, around 8–26 per fascicle; chaetiger 11 with around 25 notopodial bilimbate capillaries; and neuropodia with 5–12 hooded hooks per fascicle (Fig. 2E–G, J). Hooded hooks with several rows of subapical teeth above main fang, basal row with 3–5 teeth, and apical one multidentate, smooth hood (Fig. 2E). Notopodia dorsolaterally inserted in first four thoracic chaetigers, then gradually located more dorsally (Fig. 2A). Neuropodia ventrolateral. Lateral organs present along body, positioned between noto- and neuropodia; thoracic lateral organs oval, close to notopodia (Fig. 2C); anterior abdominal lateral organs globular, exposed (Fig. 2H). Genital pores on last thoracic chaetigers, located on intersegmental areas of chaetigers 8/9, 9/10, 10/11, and 11/12 (Fig. 2A). Transition between thorax and abdomen marked by chaetal change and size of segments (Fig. 2D, G). Abdominal chaetigers with smooth epithelium and hooded hooks on both rami (Fig. 2F, H). Abdominal hooks of similar shape to thoracic hooks but shaft shorter (Fig. 2I). Notopodial lobes close together on anterior abdominal region, chaetal fascicles with 10–16 hooded hooks (Fig. 2H). Neuropodial lobes lateral, expanded up to dorsal region, ventrally separated (Fig. 2H); chaetal fascicles with around 20 hooded hooks. Notopodial and neuropodial abdominal hooded hooks of similar shape, shoulder developed and moderate hood (Fig. 2I); posterior shaft longer than anterior one. Branchiae not observed. Pygidium unknown.

##### Methyl green staining pattern.

**Figure 3.**
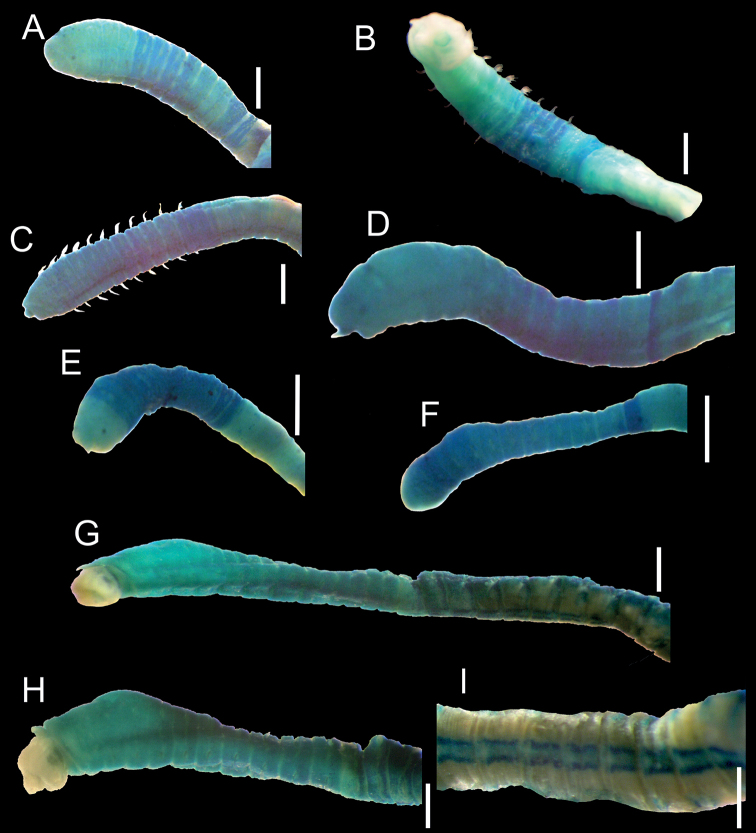
Methyl green staining patterns. *Notomastusbermejoi* sp. nov. **A** holotype (CNAP-POH-17-002) **B–E** paratypes (CNAP-POP-005 to 008) **F** additional material (CNAP-PO-036/GCA-CS-2006) **G–I***Notomastusamericanus* Day, 1973 (= *N.hemipodus* Hartman, 1945), holotype (USNM43118) **A** thoracic region, lateral view **B** thoracic and anterior abdominal regions, ventral view **C–F** thoracic and anterior abdominal regions, lateral view **G** thoracic and anterior abdominal regions, lateral view **H** anterior region, lateral view **I** abdominal region, ventral view. Scale bars: 0.5 mm (**A–F**); 1 mm (**G–I**).

Holotype with prostomium, peristomium, and chaetigers 1–4 slightly stained; chaetigers 5–10 with green bands encircling the biannulate segments, separated by an unstained ring corresponding to the fringe between chaetigers; in chaetigers 11 or 12 a darker, solid, green band encircling body (Fig. 3A). In paratypes, chaetigers 11 and/or 12 have a darker, solid, green band encircling body (Fig. 3B–E). In the additional material some thin specimens stained green from chaetiger 2 or 3 and a darker green band only on chaetiger 11 (Fig. 3F). Abdominal region uniformly stained light green.

##### Remarks.

So far, seven species of the genus *Notomastus* bearing hooded hooks on some thoracic parapodia had been accepted as valid species. They can be classified in two main groups: those species with the first chaetiger biramous and those having the first chaetiger uniramous (only notopodium present) (Table 2). In the first group we find *N.daueri*, *N.angelicae*, and *N.precocis* Hartman, 1960, from the northern Gulf of Mexico, Gulf of California, and California, respectively. The second group, with species having the first chaetiger uniramous, includes *N.teres*, *Notomastus* sp. A of Ewing, 1984a (a species still not formally named), *N.mossambicus* (Thomassin, 1970), *N.sunae* Lin, García-Garza, Lyu & Wang, 2020 and the new species *N.bermejoi* (Table 2).

**Table 2.** Comparison of *Notomastus* species with hooded hooks in thoracic chaetigers.

Species	Length	Palpode	Eyespots	Thoracic epithelium	First chaetiger	Thoracic segments	Neuropodia with hooks	Branchiae
*N.angelicae* Hernández-Alcántara and Solís-Weiss, 1998	15 mm (48 segments, incomplete)	Present	Present	1 to 4 areolated	Biramous	Biannulate	11	Not observed
*N.daueri* Ewing, 1982	65 mm (234 segments, complete)	Absent	Absent	1 to 4–5 faintly areolated	Biramous	Ventral biannulation	11	From chaetiger 60
*N.mossambicus* (Thomassin, 1970)	32 mm (105 segments, incomplete)	Absent	Present	1 to 3–4 hexagonal areolation	Uniramous	Uniannulate	11	Not observed
*N.precocis* Hartman, 1960	15.5 (around 50 segments, incomplete)	Present	Absent	Smooth	Biramous	Uniannulate	9 to 11	Posterior chaetigers
*N.sunae* Lin, García-Garza, Lyu & Wang, 2020	33.74 mm (over 100 chaetigers, complete)	Present	Present	1 to 4–5 slightly areolated	Uniramous	Biannulate	11	Not observed
*N.teres* Hartman, 1965	10.5 mm (35 segments, incomplete)	Absent	Absent	Smooth	Uniramous	Uniannulate	10 and 11	Not observed
*Notomastus* sp. A of Ewing, 1984a	4 mm (29 segments, incomplete)	Absent	Present (usually)	Smooth	Uniramous	Uniannulate	9 (mixed), 10 and 11	Not observed
*Notomastusbermejoi* **sp. nov.**	13.5 mm (34 segments, incomplete)	Present	Not observed	1 to 5–6 tessellated	Uniramous	Biannulate	11	Not observed

Initially, *N.bermejoi* sp. nov. can be clearly separated from these species, because in *N.teres* from Bermuda and New England, *Notomastus* sp. A from the northern Gulf of Mexico, and *N.mossambicus* from Madagascar the prostomium lacks an anterior palpode and their thoracic chaetigers are uniannulated. In addition, *N.teres* has hooded hooks on neuropodia of chaetigers 10 and 11, whereas in *Notomastus* sp. A, recognized as close to *N.teres* by Ewing (1984a), the hooded hooks are also present in neuropodia of chaetigers 10 and 11 but also, in neuropodia of chaetiger 9, capillaries and hooks are mixed (Table 2). *Notomastusmossambicus* has only hooded hooks in notopodia of chaetiger 11, but chaetigers 1–10 bear capillary chaetae of two types: one limbate and the other shorter, widely limbate, a character only observed in this species (Thomassin 1970; Çinar 2005).

*Notomastusbermejoi* sp. nov. is close to *N.sunae* from southern China, since both species have an anterior palpode in the prostomium and the neuropodia of chaetiger 11 bear hooded hooks. However, in *N.sunae* the first 4 or 5 chaetigers are faintly areolated, and mainly display a unique stained pattern: thorax pigmented blue with different intensity and abdomen with a paired stripe of ventral stain, as those observed in *N.hemipodus*, but with a very dark blue colour on dorsum (Lin et al. 2020), which is clearly different from that observed in *N.bermejoi* sp. nov. (Table 3).

**Table 3.** Methyl green staining pattern, depth and type locality of *Notomastus* species bearing hooded hooks in thoracic chaetigers.

Species	Methyl green staining pattern	Depth	Type locality
*N.angelicae* Hernández-Alcántara and Solís-Weiss, 1998	Chaetigers 1–2 medium green, 3–11 dark green	28.6 m	Sinaloa, Gulf of California
*N.daueri* Ewing, 1982	Chaetigers 3–6 medium green, 7–11 dark green	5.6–33.5 m	Louisiana, Northern Gulf of Mexico
*N.mossambicus* (Thomassin, 1970)	—	50–70 m	Madagascar
*N.precocis* Hartman, 1960	Chaetigers 1–8 dark green, 9–11 medium green with circle dark green bands	1305 m	Santa Catalina Basin, California
*N.sunae* Lin, García-Garza, Lyu & Wang, 2020	Thorax blue stained with different intensity; abdomen with a paired stripe of ventral stain, very dark blue colour on dorsum	Intertidal to 23 m	Xiamen Bay, Southern China
*N.teres* Hartman, 1965	Chaetigers 2–10 medium green with dark green bands, 11 dark green	500–4667 m	Bermuda; New England, USA
*Notomastus* sp. A of Ewing, 1984a	—	19–60 m	Off Texas, Northern Gulf of Mexico
*Notomastusbermejoi* **sp. nov.**	Chaetigers 1–4 slightly stained; chaetigers 5–10 with green bands encircling the segments, chaetigers 11–12 with a darker, solid, green band encircling body	32–106.4 m	Gulf of California

In contrast, Day (1973) described *N.americanus* from material collected in Beaufort, North Carolina, which also had a uniramous first chaetiger and hooded hooks in the neuropodia of chaetiger 11. However, earlier, Hartman (1945) had also described *N.hemipodus* from the same locality with only capillary chaetae in all thoracic chaetigers. The re-examination of the type material of both species, carried out by García-Garza et al. (2012), revealed similarities in their thoracic epithelial texture, a uniramous first chaetiger, an anterior palpode on the prostomium, and mainly the same methyl green staining pattern. Therefore, they reallocated *N.americanus* as a junior synonym of *N.hemipodus*.

Thus, the characters of these capitellid species with hooks in thoracic neuropodia are clearly different from those observed in *N.bermejoi* sp. nov., in which an anterior palpode is present in the prostomium, the thoracic chaetigers are biannulate, a tessellated epithelium is present in the peristomium and in chaetigers 1 to 5–6, all thoracic capillaries are bilimbate, the hooded hooks are present on neuropodia of chaetiger 11 (only one specimen also had hooks in neuropodia 10), and its body pigmentation displayed a pattern not observed in other species: chaetigers 1–4 slightly stained, chaetigers 5–10 with green bands encircling the segments, and chaetigers 11–12 with a darker, solid, green band encircling body (Table 3).

##### Habitat.

At depths of 32–106 m, in bottoms with 62–96% fine sand. Temperature: 13.2–17.5 °C; salinity: 34.99–35.51 psu; dissolved oxygen: 0.80–4.93 ml/L; organic carbon: 3.0–7.2% (Table 1).

##### Distribution.

*Notomastusbermejoi* sp. nov. was collected in the eastern Gulf of California shelf, from Tepoca Cape to Santa Maria Bay, and in the western Gulf, it was found in Santa Ines Bay and San Marcial Point (Fig. 1).

## ﻿Discussion

The specimens assigned to this new species were collected on the continental shelf of the Gulf of California and were originally identified as *N.americanus* due to the key character “presence of neuropodial hooded hooks on the last thoracic chaetiger” (Hernández-Alcántara and Solís-Weiss 1998). However, after a detailed revision of these specimens, their misidentification was suspected, since they displayed a clearly different methyl green staining pattern than observed in the type material of *N.americanus* (Table 3), confirming they are actually different species.

The examination of the methyl green pattern in the holotype of *N.americanus* (Fig. 3G–I) also showed that it corresponds to that found in *N.hemipodus* Hartman, 1945: chaetigers 1–6 with the same green intensity, a wide continuous longitudinal line on the ventral side from peristomium to chaetiger 6 (Fig. 3G, H) and the characteristic ventral abdominal region with a pair of longitudinal bands to the end of the body (Fig. 3I), even though *N.hemipodus* has only capillary chaetae in all thoracic chaetigers. The methyl green staining pattern of the holotype of *N.americanus* is illustrated here for the first time, which confirms the observations made by García-Garza et al. (2012) about the synonymy of both species. As García-Garza et al. (2012) had already indicated, the specimens of *N.americanus* examined by Day (1973), with hooks in neuropodia of chaetiger 11, can be considered juveniles. This was also mentioned by Ewing (1984a), when he examined specimens from the northern Gulf of Mexico, since in small specimens of *N.americanus* a mixture of capillaries and hooks in neuropodia of chaetiger 10 may also be present.

In the family Capitellidae, changes in chaetal structure during ontogeny represent a fundamental taxonomic problem, not only to identify the specimens to the genus level, but also to detect immature individuals. In *Notomastus*, as in several capitellid genera, during the chaetal development process, the hooks are gradually replaced by capillaries, so that even a mixture of hooks and capillaries can be found in neuropodia of middle and posterior thoracic segments (Ewing 1984b). However, from the 44 described species in this genus, including the new species described here, only eight have been reported with hooded hooks on some thoracic parapodia: *N.mossambicus*, *N.sunae*, *N.teres*, *N.daueri*, and *Notomastus* sp. A of Ewing (1984a), *N.precocis* Hartman, 1960, *N.angelicae*, and *N.bermejoi* sp. nov.

The first species described with hooded hooks in thoracic chaetigers was *N.precocis* by Hartman (1960), based on an incomplete specimen 15.5 mm long with nearly 50 segments, bearing a mixture of capillaries and hooded hooks in neuropodia of chaetigers 7 to 11, which was a distinctive character to separate it from close species. However, re-examination of its holotype showed that it could be an immature specimen of a known species, since the mixture of capillaries and hooks begins on chaetiger 7. When thoracic hooks in other species are present, it seems that the occurrence of hooks in neuropodia of chaetiger 11 is a stable character: *N.teres* described by Hartman (1965) from an incomplete specimen 10.5 mm long and 35 segments, showed that hooded hooks were present in neuropodia of chaetigers 10 and 11. Likewise, *N.mossambicus*, described from an incomplete individual 32 mm long and 105 segments, has hooded hooks in neuropodia of chaetiger 11. Ewing (1982) described *N.daueri* from a complete specimen, 65 mm long with 234 segments, also bearing hooks in neuropodia of chaetiger 11. Later, Ewing (1984a) described *Notomastus* sp A from an incomplete individual 4 mm long with 29 segments, with a mixture of capillaries and hooks in neuropodia of chaetiger 9, and only hooks in neuropodia of chaetigers 10 and 11. Hernández-Alcántara and Solís Weiss (1998) described *N.angelicae* from an incomplete specimen 15 mm long with 48 segments, bearing hooded hooks in neuropodia of chaetiger 11. It is important to emphasize that these authors examined 43 specimens, in which the occurrence of hooks on neuropodia of chaetiger 11 was constant, and only in one of them were capillaries and hooks in neuropodia 10 and 11 observed. Finally, in *N.bermejoi* sp. nov., where the holotype is an incomplete individual, 13.5 mm long with 32 segments, the hooded hooks are present in neuropodia of chaetiger 11 of all paratypes and additional material, except for one individual where mixed capillaries and hooks in neuropodia 10 were observed.

The occurrence of hooded hooks in thoracic neuropodia had already been discussed by Ewing (1982), who observed that immature specimens of *N.hemipodus*, *N.lobatus* (= *Rashgualobatus* (Hartman, 1947)), and *N.daueri* bear only hooded hooks or a mixture of capillaries and hooks in as many as five posterior thoracic neuropodia and rarely in 1–2 notopodia. So, he suggested that the replacement of hooks by capillary chaetae in thoracic neuropodia in *Notomastus* follows this pattern: juveniles have only hooks in several neuropodia of the posterior half of the thorax; as the specimen grows, hooks are lost (shed, broken or resorbed?) and replaced by capillaries emerging from the superior region of the chaetal fascicle. This goes on until all hooks are replaced by capillaries and the process continues towards the posterior thoracic chaetigers until the adult stage is reached. In the same study, Ewing (1982) observed several variations in the chaetal arrangement of numerous juveniles of *N.daueri*: neuropodia of chaetigers 7–11 may have only capillaries, mixed chaetal fascicles, or only hooded hooks, but chaetigers 10 and 11 were rarely found with a combination of capillaries and hooks.

Therefore, and also in accordance with the observed characters in *N.bermejoi* sp. nov. and the previous observations provided regarding other species, we can establish that in the genus *Notomastus*, though variations in the chaetal arrangement are present in several thoracic neuropodia, the presence of exclusively hooded hooks in neuropodia of chaetiger 11 is constant, and it can thus be considered as a stable character to differentiate species.

Although other localities have not yet been well explored, until now, the *Notomastus* species bearing hooded hooks in thoracic chaetigers have almost entirely been recorded in the American seas. From the eight species described with this morphological characteristic, only *N.sunae* from southern China and *N.mossambicus* from Madagascar are reported from other regions. Three species were reported from the Gulf of Mexico or northwestern Atlantic, and in the Eastern Pacific, *N.precocis* was collected in the deep Santa Catalina Basin, California, while *N.angelicae* and *N.bermejoi* sp. nov. were recorded from the continental shelf of the Gulf of California.

### ﻿Taxonomic key to species of *Notomastus* with hooded hooks in thoracic chaetigers

**Table:** 

1	First chaetiger biramous	**2**
–	First chaetiger uniramous	**4**
2	Prostomium without anterior palpode; last thoracic neuropodia with a mixture of capillaries and hooks	***N.daueri* Ewing, 1982**
–	Prostomium with an anterior palpode	**3**
3	Last 2–3 thoracic neuropodia with a mixture of capillaries and hooks; thoracic epithelium smooth	***N.precocis* Hartman, 1960**
–	Only last thoracic neuropodia with hooks; epithelium clearly areolated in first 4 thoracic chaetigers	***N.angelicae* Hernández-Alcántara & Solís-Weiss, 1998**
4	Prostomium without an anterior palpode; thoracic chaetigers uniannulated	**5**
–	Prostomium bearing an anterior palpode; thoracic chaetigers biannulated	**7**
5	Thoracic epithelium smooth; with hooks in last 2 or 3 thoracic neuropodia	**6**
–	Thoracic epithelium areolated in first 3 or 4 chaetigers; with hooks only in neuropodia 11	***N.mossambicus* (Thomassin, 1970)**
6	Neuropodia of chaetigers 10 and 11 with hooded hooks and neuropodia 9 only with capillaries	***N.teres* Hartman, 1965**
–	Neuropodia of chaetigers 10 and 11 with hooded hooks and neuropodia 9 with mixed capillaries and hooks	***Notomastus* sp. A of Ewing, 1984a**
7	First 4 or 5 chaetigers faintly areolated; body pigmentation: thorax pigmented blue with different intensity, abdomen with a paired stripe of ventral stain	***N.sunae* Lin, García-Garza, Lyu & Wang, 2020**
–	First 5 or 6 chaetigers tessellated; body pigmentation: chaetigers 1–4 slightly stained, chaetigers 5–10 with green bands encircling the segments, chaetigers 11 and 12 with a darker, solid, green band encircling body	***N.bermejoi* sp. nov.**

## Supplementary Material

XML Treatment for Notomastus

XML Treatment for Notomastus bermejoi
